# “We are taking every precaution to do our part…”: a comparative analysis of nursing, palliative and hospice care facilities’ websites during the COVID-19 pandemic

**DOI:** 10.1186/s12913-021-06586-y

**Published:** 2021-06-16

**Authors:** Sabahat Ölcer, Mohamed Idris¹, Yüce Yilmaz-Aslan, Patrick Brzoska

**Affiliations:** 1 Faculty of Health, School of Medicine, Health Services Research, Witten/Herdecke University, Alfred-Herrhausen- Straße 50, D-58448 Witten, Germany; 2 School of Public Health, Department of Epidemiology & International Public Health, Bielefeld University, 33501 Bielefeld, Germany; 3 School of Public Health, Department of Health Services Research and Nursing Science, Bielefeld University, 33501 Bielefeld, Germany

**Keywords:** COVID-19, Patient-focused health care services, Public awareness, Emotional resilience, Ethical guidelines, Pandemic agenda

## Abstract

**Background:**

The COVID-19 pandemic has a significant impact on health care processes. Precautions such as restrictions imposed on visitors and social distancing have led to multiple challenges, particularly in terms of communication. Using the case of nursing, palliative and hospice care facilities in Germany and drawing on agenda-setting theory, the present study aims to shed light on how health care facilities use their websites to inform (potential) health care users about changes in regulations, new protective measures implemented and about recommendations in the context of the COVID-19 pandemic.

**Methods:**

The websites of nursing, palliative and hospice care facilities in Germany were examined using qualitative document analysis and qualitative content analysis. A total of 138 websites was analysed in the study. The data gathered includes all information about COVID-19 on these websites published from the beginning of March until August 15, 2020.

**Results:**

Facilities show similarities in adhering to the measures taken by the authorities to restrict the spread of SARS-CoV-2 and to protect vulnerable patients and employees. All facilities urged the public to avoid paying visits to patients in facilities unless there was an emergency; however, visiting procedures in practice varied by types of facilities. For optimal communication, telephone and especially video calls were the options preferred by health care providers and health care users. Facilities made great efforts to prevent emotional stress and to strengthen resilience among all stakeholders. Transparency was adopted by many facilities in order to build the public’s trust.

**Conclusions:**

The agenda of health care facilities has been seriously affected by the COVID-19 pandemic. The study sheds light on the strategies developed by facilities, their efforts to increase emotional resilience among health care staff and health care users, the ethical guidelines they have adopted regarding privacy policies as well as how these themes are communicated via the facilities’ websites. The results can inform other health care facilities about how websites can be used as essential communication tools in times of public health crises.

## Background

Countries have been affected by the COVID-19 pandemic at different levels, and the precautions taken by them have also differed in terms of their general characteristics resulting from normative regulations and cultural values. The number of cases still shows marked fluctuations for each region. With some exceptions, the general trend in most countries, including Germany [1], was a dramatic increase from the second week of March to the end of April 2020 [2]. During that time interval the major goal of the authorities was to reduce the number of infections in the population in order to limit the pressure on the health care systems [3]. After the spread of the virus was – at least temporarily – brought under control, a steady decline in confirmed cases was observed [2]. Following this period of ‘normalization’, however, starting in October/November 2020, the incidence of infections showed considerable growth in many countries, creating concern among both the public and the authorities, and renewing debates on the re-enactment of protective measures.

The COVID-19 pandemic significantly affects everyday life and has a particular impact on health care services. This includes the need to increase the capacity for COVID-19 patients, the adaptation of operational procedures, the postponement of individual diagnostic measures and therapeutic interventions [4]. The primary objective of health care facilities has been to maintain adequate and patient-centered health care as best as possible, while implementing measures to ensure the wellbeing and safety of their employees and patients [5, 6]. The ban on visitors to health care facilities is a common regulation imposed by many authorities, along with social distancing and hygiene measures. However, precautions such as visiting bans and social distancing have led to multiple difficulties and limitations, which also put high demands on the *communication* between the health care facilities and health care users. The high load of information with respect to the spread of SARS-CoV-2, the symptoms of COVID-19, and rapid changes in regulations in response to daily developments have created the need and expectation for *up-to-date information* provided by respective facilities. Confronting everyday practices that are drastically unfamiliar [7] has led to the emergence of emotional stress, especially in vulnerable populations such as the elderly and those with chronic diseases. Health care professionals have similarly tried to cope with emotional stressors, such as the increased risk of exposure to the virus, extreme workloads, and moral dilemmas [8].

Taken as a whole, innovative measures/approaches [9] have become necessary to support optimal communication, while reducing the risk of direct person-to-person contact. The websites of health care facilities can serve that purpose by providing health care users and their relatives with COVID-19-related health information and practical guidelines. In that respect, health care providers can be regarded as potential mediators for the presentation of up-to-date health-related information among stakeholders in times of public health crises. Regional and national authorities are indirectly decisive in the choice of website content. Regarding this content, the preferences of the facilities to abide by the recommendations and regulations of the authorities can be seen as *mandatory volunteerism*, because the pandemic itself as a global threat constitutes one of the most important factors affecting the *decision-making mechanisms* of facilities in determining steps to be taken and the measures to be communicated.

Little is known about how health care facilities use their websites as media instruments to inform health care users about necessary changes in health care processes and regulations. Using the case of nursing, palliative and hospice care facilities in Germany as an example and drawing on agenda-setting theory [10, 11], the present study aims to shed light on how health care facilities, by means of their websites, inform (potential) health care users about recommendations as well as about new protective measures implemented in the context of the COVID-19 pandemic. It seeks to unfold the strategies developed by facilities providing different types of health services at the institutional level and intends to explore how health facilities have approached the rights and concerns of health care users and staff. Considering that the COVID-19 pandemic has affected facilities to different degrees corresponding to the type of service they provide, this study further aims to reveal in which aspects the pandemic agenda shows similarities or distinct differences between facilities and how these are reflected on the websites.

## Methods

### Study design and data Selection

Websites from inpatient/outpatient nursing, palliative and hospice care facilities in the three German federal states of Bavaria, North Rhine-Westphalia and Mecklenburg-Vorpommern were selected through Google Search and Yahoo Search for subsequent document analysis [12, 13]. Bavaria and Mecklenburg-Vorpommern were selected as the regions most and less affected by the pandemic at that time, August 4, 2020, respectively [14] (see Table 1). North Rhine-Westphalia was chosen as the state with the highest number of inhabitants in Germany [15].

**Table 1:** Percentage of COVID-19 cases by federal states in Germany as of August 4, 2020

Federal States	Population (2020)^a^	Total Cases^b^	%
Baden-Württemberg	11 100 394	37 455	0.34
Bavaria	13 124 737	51 279	0.39
Berlin	3 669 491	9 367	0.26
Brandenburg	2 521 893	3 579	0.14
Bremen	681 202	1 787	0.26
Hamburg	1 847 253	5 444	0.29
Hesse	6 288 080	12 199	0.19
Mecklenburg-Vorpommern	1 608 138	880	0.05
Lower Saxony	7 993 608	14 634	0.18
North Rhine-Westphalia	17 947 221	49 727	0.28
Rhineland-Palatinate	4 093 903	7 583	0.19
Saarland	986 887	2 886	0.29
Saxony	4 071 971	5 548	0.14
Saxony-Anhalt	2 194 782	2 034	0.09
Schleswig-Holstein	2 903 773	3 494	0.12
Thuringia	2 133 378	3 385	0.16

Sources:^a^Gesundheitsberichterstattung des Bundes. Population by sex and age [cited 2020 04 August]. Available from: http://www.gbe-bund.de

^b^Robert Koch Institute. COVID-19: Fallzahlen in Deutschland und weltweit: Fallzahlen in Deutschland, Stand: 04.08.2020 [cited 2020 04 August]. Available from: https://www.rki.de/DE/Content/InfAZ/N/Neuartiges_Coronavirus/Fallzahlen.html

**Fig. 1:**
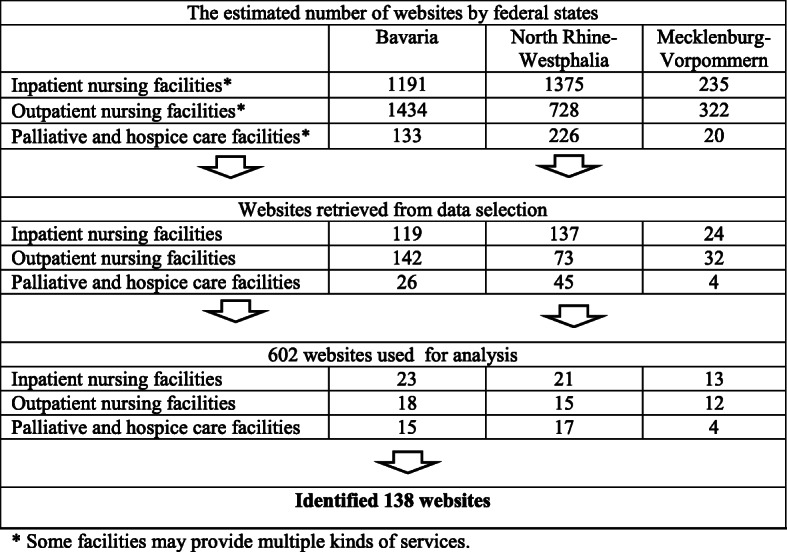
Diagram of the website identification process used in the present study

The data of the study contains all documents (i.e., information notes, news of the facilities in the press, videos, images, etc.) related to COVID-19 shared on these websites from the beginning of March until 15 August 2020. According to the initial search from the two web search engines, the total number of facilities was *N* = 5664 (see Fig. 1). In cases in which facilities provide multiple kinds of health care services such as outpatient and inpatient services, the website were considered only once for the analysis. Systematic random sampling was used, and 10 per cent of the websites were selected. After a random number between one and ten was chosen from the lists, websites were retrieved from the identified website lists, with a skip interval of 10. However, during the analysis, the likelihood of encountering COVID-19-related information on the websites of outpatient nursing and palliative and hospice care facilities was lower than for inpatient nursing facilities. Whilst this situation did not cause any problems regarding outpatient nursing facilities, it led to an increase in the sample size for palliative and hospice care facilities. Therefore, as an exception due to the small number of facilities in the three federal states, palliative and hospice care facilities were taken from the identified websites list with a skip interval of 5. Data selection continued until the saturation of the data was achieved. Websites identified were saved locally using the WinHTTrack software [16, 17]. The content of the websites was mostly in German, selected content was translated into English to be used for quotations included in the present article.

### Data Analysis

The qualitative data analysis software MAXQDA [18] was used to examine the PDF documents generated for each facility, and to encode the data. The facilities’ websites were examined using qualitative content analysis [19]. Regulations offered to the general public by the official authorities which were only reproduced by the facilities, such as documents published by the German Ministry of Health and the Robert Koch Institute, were not considered for the analysis. Websites’ content and COVID-19-related pages varied according to the types of facilities. The number of COVID-19-related pages in palliative and hospice care and inpatient nursing facilities was higher than in outpatient nursing facilities.

In the study, a total of 138 websites was analysed (see Fig. 1). The textual data were analysed using conventional qualitative content analysis, an approach with inductive category coding [19, 20]. Subsequently, based on the research questions, the main categories and sub-categories were inductively derived from the codes, with additional themes and sub-themes being identified to increase the depth of the analysis. Definitions and anchor examples were provided for each category. To strengthen the internal credibility and minimise the inconsistencies of the selected data, all codes, categories, and themes were checked and evaluated by all authors. The differences that emerged during the review and evaluation were discussed by all authors to maintain consistency, and final codes, categories, and themes were created.

### Ethical Issues

During the analysis, the abbreviations of *In* for inpatient nursing facilities, *Out* for outpatient nursing facilities, and *PalHos* for palliative and hospice care facilities were used as a standardised identifier to ensure anonymity of the facilities. These abbreviations are also used in the quotations below to indicate the type of facility. The study was approved by the responsible ethics committee of Witten/Herdecke University (No. 153/2020).

## Results

A total of three themes, with a different number of sub-themes was identified (Table 2).

**Table 2:** Themes and sub-themes generated from the information obtained from the 138 websites of nursing, palliative and hospice facilities in the German federal states of North Rhine-Westphalia, Mecklenburg-Vorpommern and Bavaria

Themes	Subthemes	Inpatients	Outpatients	Palliative and hospice care
**The strategies developed at the institutional level**	Official regulations	• Compliance with the regulations of local and regional authorities • General hygiene measures and rules • Ban on visits to health care facilities	• Compliance with the regulations of local and regional authorities • General hygiene measures and rules	• Compliance with the regulations of local and regional authorities • General hygiene measures and rules • Ban on visits to care facilities
	Reassured the vulnerable	• Protection of employees and outpatients • Medical supplies and equipment • Establishment of a crisis team evaluating the current situation on a daily basis • Recruiting additional staff • Cancellation of all events • Taking steps based on the evaluation of daily developments • Postponement of individual diagnostic measures and therapeutic interventions at short notice, except for urgent operations	• Protection of employees and outpatients • Taking steps based on the evaluation of daily developments • Recruiting additional staff • Continuity in care service but restrictions on daily routines	• Protection of employees and patients • Taking steps based on the evaluation of daily developments • Recruiting additional staff • Cancellation of activities and courses • Continuity in emergency medical operations
	Circulating up-to-date information	• Sharing up-to-date information between health care providers and stakeholders	• Sharing up-to-date information between health care providers and stakeholders	• Sharing up-to-date information between health care providers and stakeholders
	Technology-supported tools for communication	• Options for communication and counselling	• Options for communication and counselling	• Options for communication and counselling
	Clarified COVID-19-related information	• General information and FAQs (multilingual) • Links for further information	• General information and FAQs • Links for further information	• General information and FAQs (multilingual) • Links for further information
**Emotional booster efforts**		• Appreciation of employees (to increase motivation) • Calls for emotional solidarity • Organisation of some activities for employees and inpatients • Supports from local and regional companies • Continuity in religious services • Accessibility of a pastor by telephone	• Appreciation of employees (to increase motivation) • Calls for emotional solidarity	• Appreciation of employees (to increase motivation) • Calls for emotional solidarity • Supports from local and regional companies • Continuity in religious services • Accessibility to a pastor by telephone
**Ethical guidelines for the pandemic agenda**	Technology-aided health monitoring	• Transparency • Anonymised data or data logging for less than three months	• Transparency • Anonymised data or data logging for less than three months	• Transparency • Data logging for less than three months
	Supporting the grieving	• Special arrangements for visiting patients in exceptional cases • Decision making about seriously ill patients		• Special arrangements for visiting patients in exceptional cases • Decision-making about seriously ill patients

### Strategies developed at the institutional level

#### Official regulations

The pandemic agenda focused on concrete steps regarding how to slow down the spread of SARS-CoV-2 and to alleviate the pressure on health care services. Among all the facilities sampled, there was a consensus on compliance with the regulations of regional and federal authorities, especially those concerning the restrictions on physical contact and hygiene measures. Given the concerns over the rapid spread of SARS-CoV-2, possible alarming predictions led to tougher measures and new strategies to protect patients, visitors, and health care staff. Restrictions of visits to health care services were the second issue emphasised by the authorities, directly establishing the pandemic agenda of the facilities:

“Excerpt from the general decree [*referring to the enforcement announced by the Ministry of Health and Care of one of the states*]: ‘Visiting inpatient care facilities, retirement homes, and senior citizens’ residences is prohibited.*’*” (In-3).

However, restrictions varied with the type of facility. While almost no information about restrictions imposed on visitors were found on the websites of outpatient nursing facilities, inpatient nursing and palliative and hospice care facilities differed from each other depending on their service areas. Palliative and hospice care facilities throughout the pandemic were subject to relatively severe restrictions regarding visitors. By contrast, based on assessments of daily developments, visits of relatives/friends to inpatient nursing facilities varied from being severely restricted in March and April 2020 to rules being loosened until August 2020.

There were different regulations for visits inside and outside of the nursing facilities. Possible rules mentioned on the websites of the facilities included, among others, mandatory pre-registration two days in advance of the visit; mandatory completion of a registration form before the visit; use of a mobile app to register for the visit; meetings with relatives to take place in designated areas only; disinfection of hands, wearing of masks, social distancing throughout the visit and limitations in communication with nursing staff.

#### Reassured the vulnerable

According to the pandemic agenda as reflected on the websites, all facilities acted upon their evaluations of daily developments and developed short-term solutions to minimise the impact of the COVID-19 pandemic. The first common goal shared by the facilities was the protection of particularly vulnerable patients, to guarantee the wellbeing of their employees, and to provide nursing staff with intensive training in response to current circumstances:

“The health, safety, and wellbeing of our customers and employees are our top priorities.

Rest assured that we are taking every precaution to do our part to protect our customers and our employees.” (Out-7).

Palliative and hospice care facilities expressed a noticeably higher emphasis on safety measures in their agenda compared to inpatient and outpatient nursing facilities.

A second goal of facilities that emerged from the websites was to adapt to everyday practices that were drastically different from what they were familiar with. The roadmap for inpatient nursing facilities to tackle the COVID-19 pandemic consisted of the establishment of crisis teams evaluating the current situation on a daily basis. In order to protect existing patients and employees, and also considering the expansion of current capacity to meet the need for treating potentially increasing numbers of COVID-19 patients, the availability of medical supplies and equipment were other important aspects for inpatient nursing facilities. The facilities also shared the same goal regarding the recruitment of additional staff to fill gaps resulting from staff reporting sick, to reduce their employees’ workload, and to ensure the continuity of their services. Individual diagnostic measures and therapeutic interventions in inpatient nursing facilities and therapeutic measures in palliative and hospice care facilities were postponed at short notice, although urgent operations continued to be carried out in both types of facilities. Moreover, all events, activities, and courses in facilities were cancelled to reduce the risk of person-to-person transmission:

“*To protect the facilities from being infected by the virus, all events, cultural programmes, further and advanced training, seminars, and business meetings at the Z [name of an inpatient nursing facility] have been cancelled right at the beginning of March until further notice*.” (In-2).

#### Circulating up-to-date information

Due to the measures taken by facilities and the rapid changes in regulations depending on daily developments, the facilities’ communication of up-to-date information was one of the first requirements to establish understanding between health care providers and other stakeholders.

“Current information for visitors and relatives:

(Status: July 1st, 2020)

Please register early and bindingly before each visit so that there is no unnecessary waiting time.

When you enter the elderly home, you enter a protected area. Please act accordingly and follow the instructions of employees. The protection of our residents and employees remains our top priority.” (In-50).

#### Technology-supported tools for communication

Avoiding face-to-face communication was a measure adopted by almost all the facilities to decrease the risk of direct person-to-person transmission. Due to the diversity of services provided, the continuity of communication between patients and their relatives was, naturally, more of a priority issue in inpatient nursing and palliative and hospice care facilities than in outpatient nursing facilities. For optimal communication, telephone and especially video calls were the options preferred by respective facilities:

“Since contact with relatives is also important for patients, we ask you to maintain it in other ways (via telephone calls and social networks).” (PalHos-22).

Live chat or video chat with clients was also preferred over in-person conversations in the facilities’ counselling offices.

#### Provision of COVID-19-related information

Some COVID-19-related information were encountered on the websites of inpatient nursing and palliative and hospice care facilities. Frequently asked questions (FAQ) pages and general information provided on COVID-19 and SARS-CoV-2 were generally related to answers to the following questions:

- How is the virus transmitted?
- What are its symptoms?
- What should be done after COVID-19 is contracted?
- What should be done in case of suspected infection?
- How can COVID-19 be avoided?
- Where can more information be found?

Relevant links for further information included the websites of municipalities, the Robert Koch Institute, the Federal Ministry of Health, and the respective State Ministries of Health. Moreover, almost all the facilities highlighted that they were taking all the appropriate precautions, based on the recommendations of the Robert Koch Institute.

### Emotional booster efforts

On their websites many facilities documented their efforts to show appreciation for their employees’ concerns, emotional stress and anxiety, as well as to increase their motivation, build emotional resilience, and to create a sense of solidarity among health care staff and health care users:

“The on-site managers will be happy to answer any questions you may have. We all hope that at some point, normality will return for our residents, patients, guests, and our employees. Until then, however, we are very grateful to everyone for having coped with this strange time so well.” (Out-25).

Plain language has been used by almost all facilities to soften the atmosphere and to avoid confusion. Some facilities supported their employees and residents by providing gifts or bonus rewards. Unlike outpatient nursing facilities, palliative and hospice care and inpatient nursing facilities reported to have been financially and morally supported by local and regional companies:

“Our employees and the board of directors of the X [*name of a palliative and hospice care facility*] support group would like to thank Y [*names of companies and organisations*] most sincerely for the large and small donations that have been paid into the account of the X support group in the past two weeks.” (PalHos-5).

Social distancing has also led to the temporary closure of places of worship, the suspension of religious gatherings, and the inability of direct contact with religious pastors for purposes of psychological and spiritual relief during stays in the facilities. Similar to the visiting regulations also new ways of spiritual assistance have been implemented. Religious services were carried out via telephone calls or video chat. They also comprised outdoor ecumenical prayer, access to pastors by telephone, remote individual grief counselling for those who have suffered bereavement, and more recently, continuity of church services with measures of social distancing implemented.

### Ethical guidelines for the pandemic agenda

Considering the choice of the content presented on the websites, the question of how the facilities have approached the pandemic agenda and cared for the rights of their patients is among the major challenging moral issues in the health care system during the pandemic. The content of the websites demonstrates that many facilities emphasized to be “transparent” when sharing current information about their facilities in order to establish the public’s trust. This information could also include, for example, the number of patients or employees who have contracted the virus, the daily death toll, and other daily developments:

“Another three residents have died in the X [*name of an inpatient nursing facility*]. The man and the two women were aged 84, 87 and 91 respectively, and had previous illnesses. […] The positive cases are all in the new building, which is completely separated from the old building.” (In-6).

#### Technology-aided health monitoring

Facilities required visitors to use an app or a registration form in order to provide personal information and information on the current health status of the visitors, to regulate the number of incoming visitors, and to simplify appointment booking processes for visitors. The purpose of these procedures was also to ensure the wellbeing of patients and employees and to enable the authorities to trace any potential infection chain:

“I agree and consent to my data being stored in connection with the currently applicable regulations concerning COVID-19 and, if necessary, being used by the clinic or the responsible health authorities to trace contact persons. The data will be stored for a maximum of three months and destroyed afterwards.” (In-13).

#### Supporting the grieving

There were special arrangements for visiting patients in exceptional cases such as their final days of life. Patients who were in inpatient nursing facilities, and particularly in palliative and hospice care facilities, benefited from these arrangements. According to the precautions taken by the authorities, the ban on patient visits could only be lifted at the discretion of the doctors treating the patient in question.

## Discussion

The three main themes and their sub-themes identified in the present qualitative study elucidate how nursing, palliative and hospice care facilities manage their COVID-19-related agendas in order to raise public awareness of measures and applicable recommendations. The websites reviewed from different types of facilities have demonstrated that official regulations, especially the restrictions concerning physical contact and hygiene measures and compliance with these regulations were the priorities creating the respective agendas. Apart from general suggestions on social distancing, the pandemic agendas focused on the restriction or bans imposed on visitors. The facilities followed detailed guidelines including the implementation of mandatory pre-registrations.

At the beginning of the pandemic, the lack of experience and the uncertainty around SARS-CoV-2/COVID-19 led to the development of short-term solutions [3] to reduce effects on health care facilities such as bans on visits to facilities. With the progress of the pandemic and the embracement of the “new normal”, facilities developed a clearer pandemic agenda, including their priorities and responsibilities towards health care providers and beneficiaries [21]. According to the websites, for example, the cancellation of all events, activities and courses was a strategy shared by all facilities, except for a few activities continuing in outpatient nursing facilities. Moreover, the websites show that recruiting additional staff was another priority shared by all facilities. The content of the roadmap can be interpreted as preserving and strengthening the existing structures while reducing unnecessary risks.

The COVID-19 pandemic had also a direct impact on the field of communication. Communication via internet-based solutions instead of personal conversation in visits and at the counselling offices [22] was preferred and adopted in all facilities in order to avoid the infection risk associated with person-to-person contact. This telemedicine-based approach is also used in other instances when there is physical distance between health care providers and patients [23]. Especially under the conditions of the pandemic, more use of telemedicine consultations is among the practical steps recommended for palliative and hospice care patients [24].

Based on the websites it could be observed that sharing up-to-date information on developments occurring in a facility was also significant in ensuring active and transparent communication between health care providers and users. Regardless of their type, facilities sought to provide reliable and detailed information about COVID-19 and SARS-CoV-2 on their websites to all stakeholders. Considering the social diversity within the populations benefiting from the services, some inpatient nursing and palliative and hospice care facilities offered multilingual COVID-19-related information. The Robert Koch Institute was generally portrayed as one of the major reliable sources and was thus recommended by facilities for following COVID-19-related information, reflecting the fact that the Robert Koch Institute was broadly regarded as the main scientific authority in Germany.

The pandemic agenda of facilities also included efforts to increase the emotional resilience of health care users and staff and to strengthen their collaboration. In order to decrease levels of emotional stress and anxiety [22], all facilities generally appealed for solidarity among health care staff and users and expressed appreciation of their employees. Using clear language was seen as an effective way of preventing emotional stress and of strengthening resilience among staff.

As the websites show, the performance of religious rituals within facilities was similarly affected by social distancing rules implemented to prevent the spread of SARS-CoV-2 – same as in the general society [25]. Religious services were initially carried out by internet-based solutions; however, some physical services, after establishing clear guidelines, were then resumed as the pandemic situation improved. Taking up online services and the use of video mobile communication can be interpreted as the adaptation of the facilities to the changing circumstances. Permanent accessibility to a religious pastor by telephone can enable the positive therapeutic practices of religion to continue in times of crisis [26].

Transparency was adopted by many facilities as an important element to build trust among the public and potential health care users. The websites indicated the apparent connection between feelings of social unity and transparency [3, 27]. Ethical approaches of the facilities were shaped by the guidelines of the authorities. Despite the severe regulations concerning the restrictions on visits, some special arrangements were available for seriously ill patients in inpatient nursing and especially palliative and hospice care facilities. This exception was at the discretion of the doctor providing the treatment; when exceptional circumstances arise, personalised decisions about visits were reported to be ensured, weighing the risks and benefits arising from visitors [28].

To the best of our knowledge this is the first study which systematically examines how providers of nursing, palliative and hospice care make use of their websites as communication tools during the COVID-19 pandemic. The study is unique in that it compares the pandemic agendas of three different patient-focused health care services and examines their websites with regard to COVID-19-related information. Its strengths comprise the theoretical approach based on agenda-setting theory, the systematic sampling process and the large number of websites analysed. Limitations of the study include its focus on facilities from only three federal states; the inferences taken from quotations from these websites cannot necessarily be generalised to the facilities in other federal states in Germany. However, we do not assume that the spectrum of communication patterns differs between the three states examined and the other states in Germany.

## Conclusions

The study contributes to a better understanding of how health information are presented on the websites of different types of facilities during the COVID-19 pandemic. It sheds light on the strategies developed by the facilities, their efforts to increase emotional resilience among health care staff and health care users, and the ethical guidelines they have adopted regarding privacy policies.

The findings of this study show that the agendas of health facilities have been seriously affected by the COVID-19 pandemic. Even if the agendas of different health facilities show similarities in the context of the pandemic, it is clear that facilities have been affected by the pandemic to different degrees, depending on the type of services they provide. This study demonstrates that the restrictions in person-to-person contact in health services has been dominant in the agendas, as circumstances have directly led to changes in visiting regulations. It further reveals that facilities sought to clearly convey that they acted in accordance with the authorities in the steps they undertook and that there is a link between the desire for social solidarity and the facilities’ demonstration of transparency to build trust among the public. The intense sharing of COVID-19-related information through websites connotes that web-based communication is considered by health care providers as an essential communication tool in times of public health crises. Future research should consider taking advantage of web-based data by giving it a more prominent place in scientific studies. It should further explore the policies these facilities will implement post-COVID-19 to prepare for possible future public health crises, and how they will differ across different federal states or countries.

## Data Availability

All websites used for the analysis are available from the corresponding author upon request.

## Abbreviations

COVID-19: Coronavirus disease 2019
RKI: Robert Koch Institute
SARS-CoV-2: Severe acute respiratory syndrome coronavirus 2
WHO: World Health Organization
